# Galangin attenuates airway remodelling by inhibiting TGF-β1-mediated ROS generation and MAPK/Akt phosphorylation in asthma

**DOI:** 10.1038/srep11758

**Published:** 2015-07-09

**Authors:** Ya-Nan Liu, Wang-Jian Zha, Yuan Ma, Fei-Fei Chen, Wen Zhu, Ai Ge, Xiao-Ning Zeng, Mao Huang

**Affiliations:** 1 Department of Respiratory Medicine, the First Affiliated Hospital of Nanjing Medical University, Nanjing, China

## Abstract

Galangin, a natural flavonol, has attracted much attention for its potential anti-inflammatory properties. However, its role in the regulation of airway remodelling in asthma has not been explored. The present study aimed to elucidate the effects of galangin on chronic inflammation and airway remodelling and to investigate the underlying mechanisms both *in vivo* and *in vitro*. Ovalbumin (OVA)-sensitised mice were administered with galangin 30 min before challenge. Our results showed that severe inflammatory responses and airway remodelling occurred in OVA-induced mice. Treatment with galangin markedly attenuated the leakage of inflammatory cells into bronchoalveolar lavage fluid (BALF) and decreased the level of OVA-specific IgE in serum. Galangin significantly inhibited goblet cell hyperplasia, collagen deposition and α-SMA expression. Lowered level of TGF-β1 and suppressed expression of VEGF and MMP-9 were observed in BALF or lung tissue, implying that galangin has an optimal anti-remodelling effect *in vivo*. Consistently, the TGF-β1-induced proliferation of airway smooth muscle cells was reduced by galangin *in vitro*, which might be due to the alleviation of ROS levels and inhibition of MAPK pathway. Taken together, the present findings highlight a novel role for galangin as a promising anti-remodelling agent in asthma, which likely involves the TGF-β1-ROS-MAPK pathway.

Airway hyperresponsiveness (AHR), inflammation and remodelling are the major characteristics of asthma; among these, airway remodelling, which occurs at a very early stage of the disease in parallel with inflammation, has gained attention due to its key role in this condition^1^. Inflammation and remodelling are considered to interact with each other, and this interaction determines the outcome of asthma. Great efforts have been directed toward the management of airway remodelling; however, few strategies have proved effective for not stopping “the remodelling clock”. Thus, there is an urgent need for new therapeutic options for airway remodelling in asthma.

Remodelling entails a wide array of pathophysiologic events such as epithelial damage, mucus gland and goblet cell hyperplasia, subepithelial fibrosis, increased smooth muscle mass (including hypertrophy and hyperplasia), and vascular changes^2^. All of these factors contribute to persistent AHR and irreversible airway obstruction with a decrease in pulmonary function. As pivotal elements involved in AHR and remodelling, airway smooth muscle cells (ASMCs) elicit a modulatory role in airway contractility and insults such as infection, allergens or environmental factors, could alter the “synthetic” profile of ASMCs, in turn impacting airway remodelling. ASMCs can produce a range of extracellular matrix (ECM) proteins, including collagen, matrix metalloproteinases (MMPs), MMPs inhibitors, pro- and anti-inflammatory cytokines, growth factors as well as angiogenic factors (e.g., vascular endothelial growth factor [VEGF]). Due to autocrine or paracrine effects, changes in the size (hypertrophy) and/or number (hyperplasia) of ASMCs can occur in asthmatics^1^. New strategies targeting the proliferation and the activity of ASMCs in the lungs may help to control airway remodelling in the management of asthma.

Several reports have suggested that transforming growth factor-β1 (TGF-β1) plays a crucial role in airways remodelling and ASMC proliferation^2^,^3^,^4^. The hyperplasia and hypertrophy of ASMCs induced by TGF-β1 is probably attributed to the generation of intracellular reactive oxygen species (ROS)^5^. An oxidant/antioxidant imbalance is thought to be the crux in the development of chronic inflammatory airway diseases, such as asthma and chronic obstructive pulmonary disease (COPD)^6^,^7^.

Galangin (3, 5, 7-trihydroxyflavone, Fig. 1A), a member of the flavonol class of flavonoids, exists in high concentrations in honey and *Alpinia officinarum*, a plant that has been used as a spice and a herbal medicine for a variety of ailments^8^. It has been demonstrated that galangin exhibits anti-inflammatory, anti-oxidant and anti-fibrotic activities in various disorders^9^. Our previous findings established that galangin could alleviate ovalbumin (OVA)-induced airway inflammation by inhibiting NF-κB pathway^10^. Recently, it was also confirmed that galangin could ameliorate liver fibrosis and CCl4-induced hepatic stellate cell activation and proliferation^9^, indicating a regulatory potential of galangin in pulmonary fibrosis and remodelling. Since the regulatory role of galangin in airway remodelling and the underlying mechanism in asthma have not yet been studied, the present study was designed to investigate the effects of galangin on airway remodelling both *in vivo* and *in vitro*.

## Results

### Effect of galangin on chronic airway inflammation

**Figure 1:**
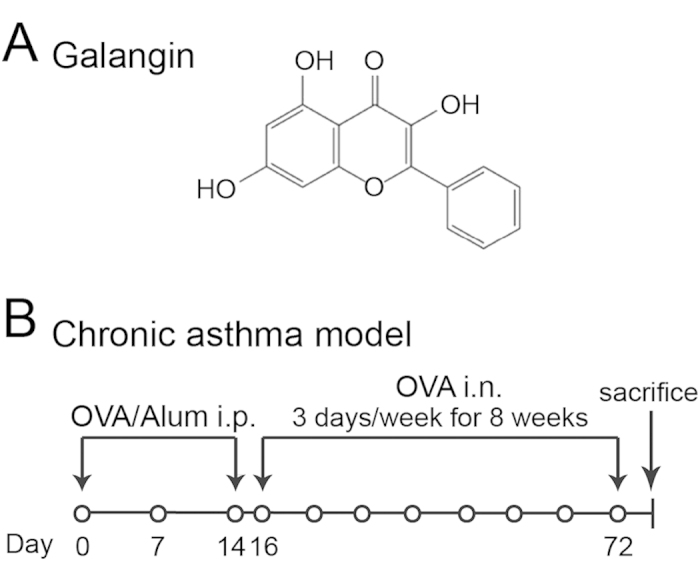
Chemical structure of galangin and experimental protocol for the chronic asthma model. (**A**) Chemical structure of galangin. (**B**) BALB/c mice were sensitised with OVA and aluminium hydroxide gel by intraperitoneal injection on days 0, 7 and 14 and then challenged with an aerosolised 5% OVA for 8 weeks beginning on the 16th day of the experiment. The mice were challenged for 30 min a day, three days a week. The control mice were sensitised and challenged with saline using the same protocol. Galangin, DEX or vehicle (DMSO) was administered via intraperitoneal injection 30 min before the challenge. OVA, ovalbumin; Alum, aluminum hydroxide gel; i.p., intraperitoneal injection; i.n., inhalation; DEX, dexamethasone; DMSO, dimethylsulphoxide.

**Figure 2:**
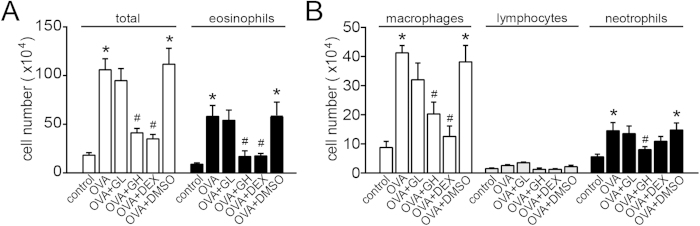
Treatment with galangin reduces the inflammatory cells trafficking into BALF. (**A,B**) Cell numbers and cell differentiation in the BALF were determined using a haemocytometer to count at least 200 cells. The data were expressed as the means ± SEM (n = 8 per group). *P < 0.05 compared with the control; ^#^P < 0.05 compared with the OVA+DMSO group. OVA, ovalbumin; GL, low dose of galangin (0.1 mg/kg); GH, high dose of galangin (0.5 mg/kg); DEX, dexamethasone; DMSO, dimethylsulphoxide; BALF, bronchial alveolar lavage fluid.

**Figure 3:**
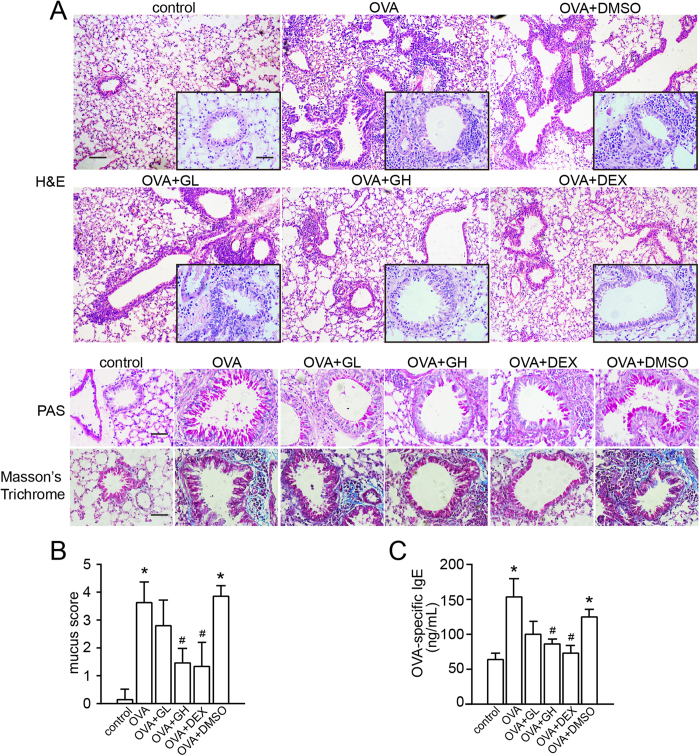
Treatment with galangin reduces inflammatory cell infiltration, goblet cell hyperplasia, collagen deposition and the level of OVA-specific IgE. (**A**) Lung sections were stained with H&E to analyse the infiltration of inflammatory cells, PAS to assess goblet cell hyperplasia, and Masson’s Trichrome to evaluate the subepithelial deposition of collagen and fibrosis. H&E,  × 100 magnification (inset  × 400 magnification), scale bar: 200 μm (inset: 50 μm); PAS and Masson’s Trichrome,  × 400 magnification, scale bar: 50 μm. (**B**) Semi quantitative analysis of mucus production in lung sections were performed as previously described^11^. Graphs represented the mucus score were expressed the means ± SEM. (**C**) The concentration of OVA-specific IgE was measured by enzyme-linked immunosorbent assay. The data were expressed as the means ± SEM (n = 8 per group). *P < 0.05 compared with the control; ^#^P < 0.05 compared with the OVA+DMSO group. OVA, ovalbumin; GL, low dose of galangin (0.1 mg/kg); GH, high dose of galangin (0.5 mg/kg); DEX, dexamethasone; DMSO, dimethylsulphoxide; H&E, haematoxylin and eosin staining; PAS, periodic acid-Schiff.

The protocols for the OVA-induced chronic asthma model are summarized in Fig. 1B. Following sensitisation and challenges, the numbers of total leukocytes as well as eosinophils, macrophages and neutrophils in the bronchoalveolar lavage fluid (BALF) were apparently increased compared to the control (Fig. 2). High dose of galangin (0.5 mg/kg) decreased the counts of total cell, eosinophil, macrophage and neutrophil to 36%, 28%, 53% and 54%, respectively, compared with the vehicle (P < 0.05). However, low dose of galangin (0.1 mg/kg) did not cause a significant difference. Lung sections were stained with haematoxylin and eosin (H&E), and inflammatory cells in BALF were counted 24 h after the last OVA challenge. Our results showed that OVA-induced mice developed severe inflammatory responses in the airways, including extensive inflammatory cell infiltration around the respiratory tract and vessels and inflammatory cell trafficking into BALF (Fig. 3A). Treatment with galangin (0.5 mg/kg) and dexamethasone (DEX) significantly suppressed the infiltration of inflammatory cells, while treatment with vehicle (OVA+dimethylphsulfoxide [DMSO] group) did not lead to improvement (Fig. 3A).

### Effect of galangin on goblet cell hyperplasia and collagen deposition/fibrosis

The number and mucus production of goblet cells were assessed by Periodic Acid-Schiff (PAS) staining. The PAS-positive cells per bronchiole were also counted in a double-blind manner using a numerical scoring system (Fig. 3B)^11^. Meanwhile, the extent of collagen deposition/fibrosis was evaluated by Masson’s trichrome stain. As shown in Fig. 3A, marked goblet cell hyperplasia and mucus hypersecretion were observed in the bronchioles of OVA-challenged mice. Compared with the vehicle, galangin (0.5 mg/kg) and DEX treatment decreased the number of goblet cells in the airway epithelium and halted the mucus hypersecretion. Collagen deposition was profoundly increased over the interstitium of the airways and vessels in the OVA and the OVA+DMSO groups (Fig. 3A). This increase in airway collagen deposition and fibrosis was reversed by high dose of galangin (0.5 mg/kg) and DEX administration (Fig. 3A).

### Effect of galangin on the level of OVA-specific IgE in serum

A key feature of bronchial asthma is the high level of IgE. After sensitisation and challenges, the level of OVA-specific IgE in serum was significantly elevated both in the OVA and the OVA+DMSO groups compared with the control, whereas this elevation was abolished by both high dose of galangin (0.5 mg/kg) and DEX administration (Fig. 3C).

### Effect of galangin on α-SMA and MMP-9 expression

**Figure 4:**
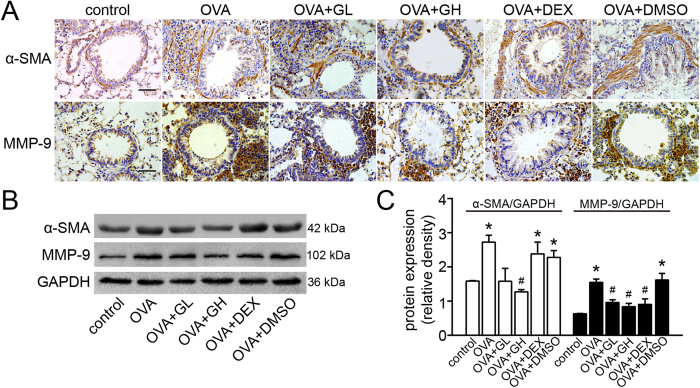
Treatment with galangin inhibits the expression of α-SMA and MMP-9 in lung tissue. (**A**) Immunohistochemical staining was performed to assess the distribution of α-SMA and MMP-9.  × 400 magnification, scale bar: 50 μm. (**B**) The expression of α-SMA and MMP-9 was analysed by western blot. GAPDH was utilised as the standard control. (**C**) The band signal strengths of α-SMA and MMP-9 were expressed as a ratio to GAPDH. The data were expressed as the means ± SEM (n = 8 per group). *P < 0.05 compared with the control; ^#^P<0.05 compared with the OVA+DMSO group. OVA, ovalbumin; GL, low dose of galangin (0.1 mg/kg); GH, high dose of galangin (0.5 mg/kg); DEX, dexamethasone; DMSO, dimethylsulphoxide; α-SMA, smooth muscle actin alpha chain; MMP-9, matrix metallopeptidase-9; GAPDH, reduced glyceraldehyde phosphate dehydrogenase.

Representative photomicrographs of immunohistochemical staining for smooth muscle actin alpha chain (α-SMA) and MMP-9 in the airways are shown in Fig. 4A. The staining of α-SMA and MMP-9 around the airways and the number of infiltrated inflammatory cells were increased in the OVA and OVA+DMSO groups compared with the control. Galangin (0.5 mg/kg) dramatically decreased the areas of α-SMA and MMP-9 staining, though no variation in α-SMA was observed in the DEX group. These observations were consistent with the results obtained from western blot (Fig. 4B,C), implying that galangin and DEX may exert their anti-remodelling effects through different mechanisms.

### Effect of galangin on the level of TGF-β1 and VEGF

**Figure 5:**
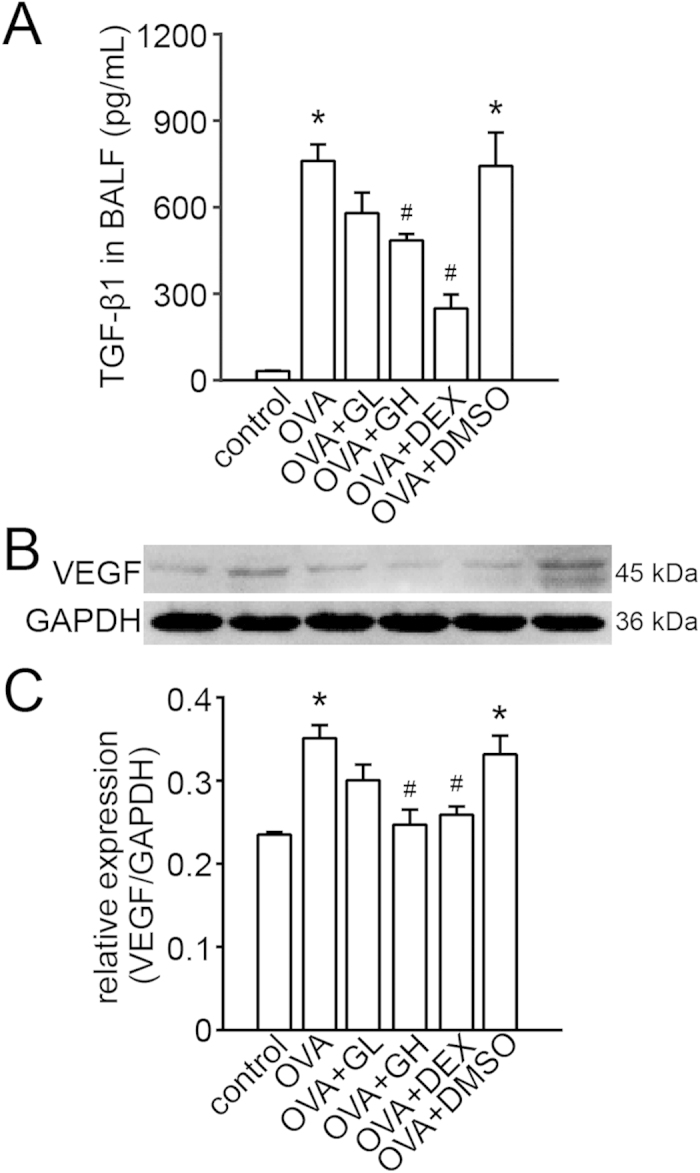
Treatment with galangin inhibits the expression of VEGF in lung tissue and decreases the level of TGF-β1 in BALF. (**A**) The concentrations of TGF-β1 were measured using an enzyme-linked immunosorbent assay. The data were expressed as the means ± SEM (n = 8 per group). (**B**) The expression of VEGF was analysed by western blot. GAPDH was utilised as the standard control. (**C**) The band signal strength of VEGF was expressed as a ratio to GAPDH. The data were expressed as the means ± SEM (n = 8 per group). *P < 0.05 compared with the control; ^#^P < 0.05 compared with the OVA +DMSO group. OVA, ovalbumin; GL, low dose of galangin (0.1 mg/kg); GH, high dose of galangin (0.5 mg/kg); DEX, dexamethasone; DMSO, dimethylsulphoxide; TGF-β1, transforming growth factor-β1; BALF, bronchial alveolar lavage fluid; VEGF, vascular endothelial growth factor; GAPDH, reduced glyceraldehyde-phosphate dehydrogenase.

TGF-β1 plays a critical role in airway remodelling. Following sensitisation and challenges, the BALF TGF-β1 levels in the OVA group and the vehicle were markedly elevated to 761.9 ± 56.7 pg/mL and 743.5 ± 116.2 pg/mL; in contrast, both high and low dose of galangin decreased the BALF TGF-β1 levels to 434.9 ± 53.6 pg/mL and 580.7 ± 70.7 pg/mL, respectively (Fig. 5A). Results obtained from western blot revealed that VEGF was up-regulated both in the OVA and the OVA+DMSO groups compared with the control. This up-regulation was almost reversed by galangin (0.5 mg/kg) and DEX (Fig. 5B,C).

### Effect of galangin on TGF-β1-induced proliferation of human ASMCs

**Figure 6:**
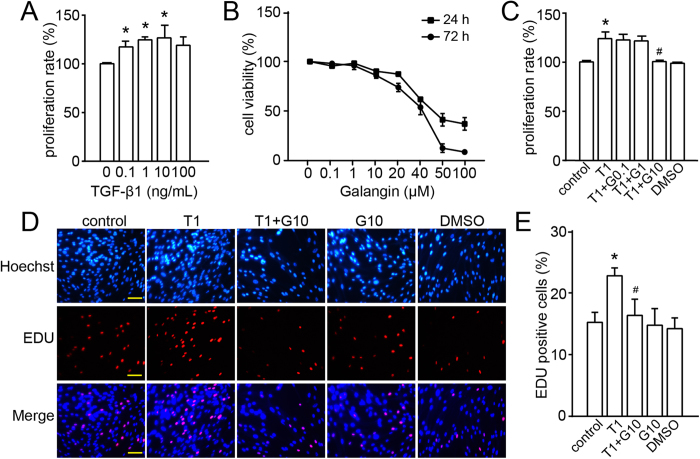
Treatment with galangin inhibits the TGF-β1-induced ASMC proliferation. (**A**) Effect of stimulation with different concentrations of TGF-β1 on the proliferation of human ASMCs as assessed by the CCK-8 assay. (**B**) Effect of galangin on the viability of human ASMCs as assessed by the CCK-8 assay. (C-E) Effect of galangin on TGF-β1-stimulated human ASMCs as assessed by the CCK-8 and EdU assays. Scale bar: 100 μm. The values were presented as the means ± SEM of three replicates. *P < 0.05 compared with the control; ^#^P < 0.05 compared with the TGF-β1 group. T1, TGF-β1, transforming growth factor-β1 (1 ng/mL); G, galangin (G0.1, 0.1 μM; G1, 1 μM; G10, 10 μM); DMSO, dimethylsulphoxide; ASMC, airway smooth muscle cell; CCK-8, cell counting kit; EDU, 5-ethynyl-2′-deoxyuridine.

The proliferation of human ASMCs was promoted by TGF-β1 stimulation (Fig. 6A). We determined the toxicity of galangin (0.1, 1, 10, 20, 40, 50 and 100 μM) on human ASMCs. The cell viabilities were 90% and 86% in the 10 μM-group at 24 h and 72 h (Fig. 6B). And galangin (0.1–10 μM) inhibited TGF-β1 (1 ng/mL, T1)-induced ASMC proliferation in a dose-dependent manner. Treatment with 10 μM galangin (G10) decreased T1-induced ASMC proliferation from 124% ± 8% to 101% ± 2% (P < 0.05); no significant effect was induced by 0.1 or 1 μM galangin (Fig. 6C). Furthermore, by using a 5-ethynyl-2′-deoxyuridine (EdU) incorporation assay^12^, we observed that the number of cells incorporating EdU was notably increased after TGF-β1 stimulation compared to the control, whereas galangin pretreatment distinctly attenuated TGF-β1-induced proliferation (Fig. 6D,E).

### Effect of galangin on TGF-β1-induced ROS generation

**Figure 7:**
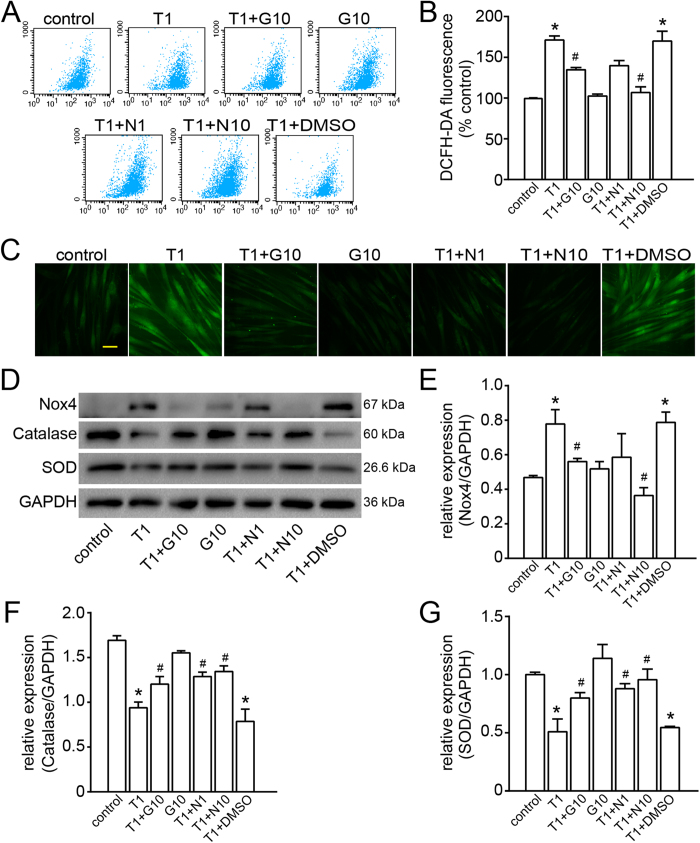
Treatment with galangin attenuates intracellular ROS production and reverses the oxidant/antioxidant imbalance after TGF-β1-induced oxidative stress. (**A,B**) Fluorescence-activated cell sorter profile of ROS generation by flow cytometry. Summary of average ROS production by ASMCs treated with galangin (10 μM) or NAC (1, 10 mM) after TGF-β1 (1 ng/mL) stimulation in three independent experiments. (**C**) DCFH-DA fluorescence (green) imaging of ROS in ASMCs using a confocal laser scanning microscope. Scale bar: 100 μm. (**D**) The levels of Nox4, catalase and SOD in ASMCs were measured by western blot. The data are expressed as folds change in protein expression normalised to GAPDH expression. (**E-G**) The band signal strengths of Nox4, catalase and SOD were expressed as a ratio to GAPDH. The data were expressed as the means ± SEM of three replicates. *P < 0.05 compared with the control; ^#^P < 0.05 compared with the TGF-β1+DMSO group. T1, TGF-β1, transforming growth factor-β1 (1 ng/mL); G10, galangin (10 μM); DMSO, dimethylsulphoxide; N, NAC, N-acetylcysteine (N1, 1 mM; N10, 10 mM); ROS, reactive oxygen species; ASMC, airway smooth muscle cell; DCFH-DA, 2´,7´-dichlorofluorescin diacetate; Nox4, NADPH oxidase 4; SOD, superoxide dismutase; GAPDH, reduced glyceraldehyde-phosphate dehydrogenase.

Since ROS may serve as signalling molecules to mediate multiple cell functions, we examined whether galangin could regulate ROS generation. As shown in Fig. 7, ROS production was promoted by TGF-β1, which is an effective activator in ASMCs^6^. Flow cytometry analysis showed that pretreatment with 10 μM galangin, 1 mM N-acetyl cysteine (NAC) or 10 mM NAC decreased the intracellular levels of ROS to 79%, 82% or 62%, respectively, compared to the vehicle control (Fig. 7A,B). This result suggests that galangin has an anti-oxidant effect similar to that of NAC (a potent ROS scavenger). The ROS levels in ASMCs were also monitored using a confocal laser scanning microscope, and galangin significantly attenuated the levels of ROS in TGF-β1-treated ASMCs (Fig. 7C).

### Effect of galangin on the oxidant/antioxidant imbalance

The oxidant/antioxidant imbalance is believed to be a key event in asthma. In the present study, we found that TGF-β1 up-regulated the expression of NADPH oxidase 4 (Nox4) while down-regulating the expression of superoxide dismutase (SOD) and catalase, which might be responsible for the trend observed in the ROS levels (as described previously). This change in oxidant/antioxidant enzymes was partially reversed by galangin and NAC (Fig. 7D–G).

### Effect of galangin on MAPK/Akt pathways in human ASMCs

**Figure 8:**
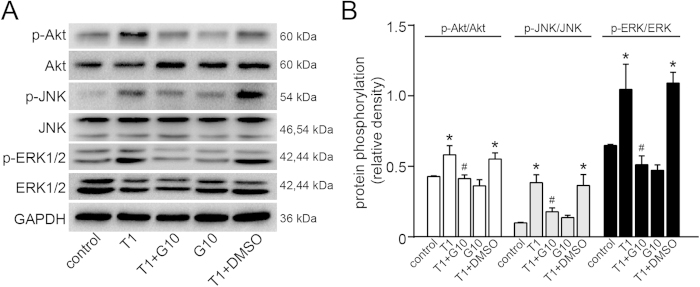
Treatment with galangin inhibited the activation of ERK, JNK and Akt pathways in ASMCs. The levels of phosphorylated and total ERK, JNK and Akt were measured by western blot. The data were reported as the means ± SEM of three replicates. *P < 0.05 compared with the control; ^#^P < 0.05 compared with the TGF-β1 + DMSO group. T1, TGF-β1, transforming growth factor-β1 (1 ng/mL); G10, galangin (10 μM); DMSO, dimethylsulphoxide; ASMC, airway smooth muscle cell; p-Akt, phosphorylated Akt; p-JNK, phosphorylated c-Jun N-terminal kinase; p-ERK, phosphorylated extracellular regulated protein kinases; GAPDH, reduced glyceraldehyde-phosphate dehydrogenase.

To determine the signalling mechanisms involved in the effects of galangin on airway remodelling, ASMC proliferation and oxidant/antioxidant enzymes, the phosphorylation status of ERK, JNK and Akt was investigated. As shown in Fig. 8, we found that TGF-β1 could activate ERK, JNK and Akt in ASMCs within 1 h after stimulation. This activation was partially blunted in the group pretreated with galangin (10 μM) compared to the vehicle control. These results suggest that galangin likely attenuates airway remodelling and ASMC proliferation by inhibiting activation of MAPK/Akt pathways.

## Discussion

Galangin, a natural flavonol, has attracted much attention for its potential anti-inflammatory and anti-oxidant properties. It has been demonstrated that galangin can reduce lipopolysaccharide-induced acute lung injury in mice by inhibiting inflammation and oxidative stress^13^, modulate immune complex-mediated neutrophil activation in rheumatoid arthritis^14^ and attenuate mast cell-mediated allergic inflammation^15^. Our previous study also showed that airway inflammation and AHR were suppressed by galangin in an acute model of asthma, indicating that galangin has effects similar to those of glucocorticoids (e.g. dexamethasone)^10^. The present study further indicated that galangin significantly decreased the OVA-specific IgE level in serum, which induces mast cell activation and leads to airway inflammation^16^, in a chronic model of asthma, suggesting that galangin has a regulatory effect on both acute and chronic asthmatic inflammation.

As the first-line agents for asthma treatment, glucocorticoids reap few fruits in the improvement of airway remodelling^17^. Remodelling, which leads to fixed airway obstruction, might contribute to the poor response of patients with refractory asthma to treatments^18^. Thus, it is extremely urgent to develop new therapeutic options for asthmatics that are safe and effective. In the present study, we established a chronic model of allergic asthma to investigate the effect of galangin on airway remodelling. As mentioned previously, goblet cell hyperplasia, collagen deposition and an increase in airway smooth muscle mass (including hypertrophy and hyperplasia) have been observed, and these processes are considered to be key events in asthmatic airway remodelling. Mucus hypersecretion could lead to airway obstruction, contributing to the morbidity and mortality of asthma^19^,^20^. Additionally, ECM components, particularly collagen, account for the formation of subepithelial fibroblasts^21^. Our results demonstrated that galangin markedly reduced goblet cell hyperplasia and collagen deposition/fibrosis, indicating that galangin may have an inhibitory role in asthmatic airway remodelling.

α-SMA is widely viewed as a contractile element in ASMCs which are involved in the progression of lung remodelling and bronchoconstrictor responsiveness. Increased levels of α-SMA and other contractile components serve to facilitate ASMC proliferation and migration, which are crucial for airway remodelling^22^. In this study, the results obtained from immunohistochemical staining and western blot showed that the level of α-SMA in airways was decreased by galangin in chronically challenged mice, whereas only modest effects were observed with DEX treated mice. It has also been reported that DEX failed to attenuate the expression of α-SMA in lung fibroblasts^23^, suggesting that galangin has an advantage over DEX with respect to suppression of ASMC hyperplasia and hypertrophy.

MMP-9 is known to be involved in collagen deposition in airway walls, leading to narrowed airways^24^,^25^. Our results indicated that galangin significantly restrained OVA-induced MMP-9 expression, contributing to the attenuation of ECM deposition and fibrosis. A recent study also showed that galangin exerts a global hepato-protective effect and significantly inhibits liver fibrosis, which was attributed to its effect on cell proliferation and collagen gene expression in hepatic stellate cells^9^. Thus, we concluded that the amelioration of ECM deposition and fibrosis by galangin in chronic asthma most likely accounts for the anti-fibrotic and anti-oxidant activity of galangin.

TGF-β1 is a key factor involved in airway remodelling: it contributes mainly to abnormal airway smooth muscle function in asthma or COPD by eliciting ASMC proliferation and hypertrophy and by prompting the release of angiogenic, fibrogenic and inflammatory mediators^6^. Furthermore, TGF-β1 expression has been shown to be increased in chronic lung diseases of humans such as asthma^26^,^27^, and recent evidence has also revealed that galangin inhibits TGF-β1 expression in rats with liver fibrosis^9^. Our study showed that galangin could significantly reduce the level of TGF-β1 in BALF, suggesting that TGF-β1 may be the key point implicated in the anti-remodelling effect of galangin. Moreover, it has also been confirmed that TGF-β1 enhances the release of VEGF by ASMCs in a time-dependent manner^28^. As an endothelial cell-specific mitogenic peptide, VEGF modulates vasculogenesis and angiogenesis, in turn contributing to airway obstruction or AHR^29^. ROS are considered to be the vital mediators of pulmonary vascular cell proliferation. It has been shown that ROS can enhance VEGF expression and activate the Ca^2+^ dependent transcription factor, NFATc3^30^,^31^, which increases the thickness of pulmonary arterial walls and promotes vascular remodelling^32^,^33^. In our study, we found that VEGF expression in lung tissue was significantly increased by challenge with OVA and that galangin dramatically attenuated this expression. These results suggest that galangin could down-regulate the VEGF level in association with TGF-β1 or ROS, indicating that galangin may have a certain level of anti-angiogenic activity.

Oxidative stress, the physiological damage that occurs due to ROS attack, has been demonstrated to affect smooth muscle contraction^34^,^35^, induce AHR^34^,^36^, and increase mucus secretion and epithelial shedding within respiratory cells^34^,^37^,^38^. Additionally, excessive ROS production in asthma may trigger enzymatic and non-enzymatic alterations, leading to an antioxidant/oxidant imbalance in respiratory airways^39^. This imbalance leads to a state of oxidative stress, contributing to the emergence and persistence of pulmonary fibrosis induced by TGF-β1^40^,^41^. A previous study indicated that the early release of ROS in response to TGF-β1 activates the Smad pathway, leading to the up-regulation of Nox4 and the down-regulation of catalase or MnSOD^6^. Our findings demonstrated that the disruption of the oxidant/antioxidant enzyme balance was abolished to a similar extent by galangin and NAC. As a free radical scavenger, NAC prevents oxidant-induced airway smooth muscle contraction^6^,^42^, ASMC proliferation and inflammatory mediator release^6^,^42^. We found that galangin could block TGF-β1-induced ASMC proliferation *in vitro*, suggesting that galangin may specifically target the TGF/ROS pathway.

As we all know, MAPK and PI3K are key components in signal transduction associated with cell proliferation and PI3K signaling pathway induces cell proliferation via the serine/threonine kinase Akt. It has been reported that dual ERK and PI3K pathways could control ASMC proliferation^43^. Burgess *et al*. even found that ERK and Akt activation were significantly greater in ASMCs from asthmatic subjects. In addition, LY294002 (the PI3K inhibitor) and U0126 (the MEK/ERK inhibitor) significantly inhibited cell proliferation in ASMCs from asthmatics^43^. And several reports have also indicated that TGF-β1 promotes ASMC proliferation via MAPK pathway^3^. By using PD184352 (the ERK inhibitor), Halwani *et al*. confirmed that inhibition of p42/p44 MAPK phosphorylation could alleviate ASMC proliferation induced by chemokine^44^. On the other hand, it has been indicated that TGF-β1 stimulates proliferation of human pulmonary artery SMCs by a redox-dependent process involving Nox4 NADPH oxidase with increased ROS which induced ERK1/2 phosphorylation^45^. In human ASMCs, Nox4 is substantially induced by TGF-β1 and TGF-β1 increases ROS production which could be abolished by the flavoprotein NADPH oxidase inhibitor DPI. Moreover, ASMC proliferation stimulated by TGF-β1 could be reversed by Nox4 siRNA or catalase, suggesting that TGF-β1-induced ASMC proliferation attributes in part to a redox-dependent mechanism^5^. Similarly, TGF-β also increases Nox4 expression and activity in human aortic SMCs. Transfection of aortic SMCs with Nox4 siRNA abolishes TGF-β-induced SMA expression^46^.

Various studies have indicated that ROS can also activate multiple factors involved in cellular proliferation, such as PI3K/Akt and MAPK. Thus ROS play a very important physiological role as second messengers^40^. The activation of isoenzymes of the PKC family drives oxysterol-generated signals through MAPK pathway, with the marked up-regulation of ERK and JNK kinases. Moreover, inhibition of NADPH oxidase activation and ROS production by various chemicals, such as cobalt protoporphyrin IX (a heme oxygenase-1 inducer) has been shown to reduce TNF-α-induced MAPKs phosphorylation in human ASMCs^47^, suggesting that Nox/ROS play a key role in mediating MAPKs activation in response to pro-inflammatory mediators^40^. Taken together, these studies demonstrate that the TGF-β1-induced ASMC proliferation may attribute to TGF-β1-mediated ROS generation and MAPK/Akt phosphorylation. Our data indicate that galangin inhibits TGF-β1-induced activation of ERK, JNK and Akt, suggesting that galangin specifically prevents ASMCs from developing a proliferative and pro-oxidant phenotype. Thus, galangin might prevent aberrant airway smooth muscle function in airway remodelling and may have clinical therapeutic benefits for asthma patients.

Taken together, we demonstrated the potential therapeutic effect of galangin in an experimental model of asthma and revealed its anti-oxidant and anti-proliferative properties in human ASMCs. Our results demonstrated that galangin could markedly attenuate the extent of chronic inflammation and airway remodelling and could furthermore reduce the level of TGF-β1 in BALF and suppress the expression of VEGF and MMP-9. Additionally, galangin inhibited TGF-β1-induced ASMC proliferation *in vitro*. The underlying mechanisms might involve the reduction of ROS levels and the inhibition of ERK, JNK and Akt phosphorylation. Taken together, our findings highlight the potential effects of galangin on airway remodelling, which most likely occur via the TGF-β1-ROS-MAPK pathway. The present study provides a promising therapeutic agent for asthma patients.

## Methods

### Animals

Specific-pathogen-free female BALB/c mice (18–22 g), aged 6 to 8 weeks, were obtained from Vital River Laboratories (Beijing, China). The mice were kept in a temperature-controlled room with 12-h light/dark cycles, and they were allowed access to food and water ad libitum. All experiments involving animals and tissue samples were performed according to the guidelines of the National Institutes of Health and Nanjing Medical University, and all procedures were approved by the Institutional Animal Care and Use Committee of Nanjing Medical University (Nanjing, China).

### Ovalbumin sensitisation/challenge protocol

Figure 1B shows a schematic illustration of the protocols. In total, 48 female BALB/c mice were randomly divided into the following 6 groups: control, OVA (Grade V; Sigma, St. Louis, MO, USA), OVA+GL (galangin 0.1 mg/kg; Sigma), OVA+GH (galangin 0.5 mg/kg), OVA+DEX (1 mg/kg; Sigma), and OVA + DMSO (0.4 μL in a total volume of 200 μL with saline, vehicle; Sigma). The mice were sensitised on days 0, 7 and 14 by intraperitoneal injection of 20 μg OVA emulsified in 2 mg of aluminum hydroxide gel (Invivo-Gen, San Diego, CA, USA) in a total volume of 200 μL. These sensitised mice were exposed to aerosolised 5% OVA in sterile saline for 8 weeks beginning on day 16 for 30 min three times a week. We placed the mice in chambers (51 × 31 × 21 cm) connected to an ultrasonic nebuliser (NE-U11B; OmronCorp., Tokyo, Japan) to obtain a whole-body inhalation system. Galangin (0.1 and 0.5 mg/kg), DEX and DMSO were administered 30 min before nebulisation. Control subjects were sensitised and challenged with saline using the same protocol. The mice were sacrificed 24 h after the last challenge, and BALF, sera, and lung tissues were collected for analysis.

### Bronchoalveolar lavage fluid and serum analysis

Briefly, the mice were anaesthetised by intraperitoneal injection of pentobarbital sodium (70 mg/kg) 24 h after the final challenge. BALF and serum samples were collected from each mouse. The total number of inflammatory cells in the BALF was determined by counting using a haemocytometer. Differential cell counts were performed with Wright’s staining on the basis of morphological criteria. The cells in the BALF were counted by two independent investigators in a single-blind study, in which at least 200 cells each from 3 different random locations were analysed using a microscope. The levels of TGF-β1 (R&D Systems, Abingdon, UK) in BALF and OVA-specific IgE (Shibayagi, Gunma, Japan) in serum were measured using commercial enzyme-linked immunosorbent assay (ELISA) kits according to the instructions provided by the manufacturer.

### Lung histology

After BALF samples were collected, a 20 mL syringe equipped with a 18 G needle was used to inject 10–15 mL PBS slowly into the right ventricle. Then the lungs were inflated with 4% paraformaldehyde under 20 cm pressure by a tracheal catheter and placed in 4% paraformaldehyde fixative for paraffin embeddness. A series of microsections (5 μm) were cut using a microtome and stained with H&E to assess inflammatory cell infiltration, PAS to quantify airway global cells and mucus production, Masson’s trichrome to visualise collagen deposition and fibrosis, and immunohistochemical staining to examine α-SMA and MMP-9 distribution. Additionally, the mucus score was performed by a blinded scorer to determine the extent of mucus production using a 5-point grading system as follows: 0, no goblet cells; 1, <25% goblet cells; 2, 25%–50% goblet cells; 3, 50%–75% goblet cells; 4, >75% goblet cells. Mucus scoring was performed in at least three different fields for each lung section^11^.

### Culture and treatment of normal human ASMCs

Normal human ASMCs were purchased from Scien-Cell Research Laboratories (Carlsbad, CA, USA). The cells were cultured at 37 °C with 5% CO_2_ in DMEM (Invitrogen-Gibco, Paisley, Scotland) supplemented with 20 U/L penicillin, 20 μg/mL streptomycin and 10% foetal bovine serum (HyClone, Logan, UT, USA). Cells between passages 4 and 8 were used for the experiments. After starvation, the cells were stimulated with 1 ng/mL TGF-β1 (Peprotech, Rocky Hill, USA) alone or along with galangin (10 μM) and further cultured for the indicated times. As a positive control, cells were treated with NAC in the same manner.

### Cell viability assay

The proliferation of ASMCs was determined using the cell counting kit-8 (CCK-8) assay and the EDU assay. ASMCs were cultured in a 96-well plate (Corning Incorporated, Corning, NY, USA) at a density of 5 × 10^3^ cells per well and treated with TGF-β1 at concentrations ranging between 0.1 and 100 ng/mL for 72 h. Then, CCK-8 solution (Dojindo Molecular Technologies Inc., Kumamoto, Japan) was added to the cell culture medium to achieve a final concentration of 10 μL/100 μL and incubated for an additional 1–2 h at 37 °C. The absorbance at 450  nm (A450) was read on a microplate reader (CANY, Shanghai, China). Similarly, the cytotoxicity of galangin and the interaction between TGF-β1 and galangin in ASMCs were examined using the CCK-8 assay. For the EdU assay, the cells were cultured via the aforementioned procedure and subsequently divided into five treatment groups: control, T1 (TGF-β1 1 ng/mL), T1+galangin (10  μM), galangin (10 μM) and vehicle (0.1 μL DMSO in 1000 mL DMEM). After 48 h of treatment, an EdU assay kit (Ribobio, Guangzhou, China) was used for labelling according to the manufacturer’s instructions. Images were obtained using a fluorescence microscope (Olympus IX71, Japan), and each sample was measured in triplicate.

### Intracellular ROS measurement

Intracellular ROS were measured using the 2′,7′-dichlorofluorescin diacetate (DCFH-DA, Sigma) assay. Briefly, 1.5 × 10^4^ cells were seeded into each well of a 6-well plate, cultured for 24 h, and subsequently exposed to galangin (10 μM), NAC (1 and 10 mM) or vehicle (DMSO) with TGF-β1 (1 ng/mL) for 24 h. The cells were then incubated with 10 μM DCFH-DA for 30 min at 37 °C in the dark. After incubation, the cells were washed twice with PBS and analysed within 30 min using FACScan (Becton Dickinson, San Jose, CA, United States) with excitation at 488 nm. The specific fluorescence signals corresponding to DCFH-DA were collected with a 525-nm band pass filter. A total of 10,000 cells were counted in each determination. Intracellular ROS production was also measured with a confocal laser scanning microscope (Zeiss LSM 5 live, Germany). After incubating with DCFH-DA, the cells were fixed with 4% paraformaldehyde for 10 min and washed 3 times with PBS before photographing. The excitation and emission wavelengths were identical to those described previously and photographs were taken. For each culture, a minimum of 5 random fields were captured.

### Western blot analysis

Total cell and lung protein extracts were lysed on ice in lysis buffer (Cell Signaling Technology Inc., Beverly, MA, USA) and subsequently centrifuged for 15 min at 14,000 rpm. The supernatant was then transferred into a fresh tube and stored at −80 °C. The total protein concentration was determined using the BCA protein assay (Thermo, Rockford, IL, USA). Denatured samples were mixed with a SDS-PAGE loading buffer and heated to 100 °C for 5 min. After electrophoresis, the separated proteins were transferred to polyvinylidene difluoride membranes (Millipore, Billerica, MA, USA) via the wet transfer method. Nonspecific sites were blocked with 5% non-fat milk in TBS Tween 20 (TBST; 25 mM Tris [pH 7.5], 150 mM NaCl, 0.1% Tween 20) for 2 h, and the blots were incubated with anti-catalase antibody (Abcam, Cambridge, UK), anti-SOD antibody (Abcam), anti-Nox4 antibody (Abcam), anti-MMP-9 antibody (Abcam), anti-α-SMA antibody (Abcam), anti-VEGF antibody (Abcam), anti-glyceraldehyde-3 phosphate dehydrogenase (GAPDH) antibody (Bioworld, Nanjing, China), anti-phospho-ERK antibody (Cell Signaling Technology Inc.), anti-ERK antibody (Cell Signaling Technology Inc.), anti-phospho-Akt antibody (Cell Signaling Technology Inc.), and anti-Akt antibody (Cell Signaling Technology Inc.) overnight at 4 °C. Goat anti-rabbit horseradish peroxidase-conjugated IgG (Cell Signaling Technology Inc.) was used to detect antibody binding. After treating the membranes with enhanced chemiluminescence system reagents (Thermo), the binding of specific antibodies was visualised using the Bio-Rad Gel Doc/Chemi Doc Imaging System and analysed using Quantity One software.

### Statistical analysis

The data are expressed as the means ± standard error of the mean (SEM). All tests were performed using Prism 6.00 (GraphPad Software, San Diego, CA, USA) and SPSS version 20 (SPSS Inc., Chicago, IL, USA). The results were analysed by one-way analysis of variance for repeated measures, followed by a Dunnett post hoc test to determine differences in multiple comparisons. The significance was set at P < 0.05.

## Additional Information

**How to cite this article**: Liu, Y.-N. *et al.* Galangin attenuates airway remodelling by inhibiting TGF-ß1-mediated ROS generation and MAPK/Akt phosphorylation in asthma. *Sci. Rep.* **5**, 11758; doi: 10.1038/srep11758 (2015).
